# Expression of Phosphatonin-Related Genes in Sheep, Dog and Horse Kidneys Using Quantitative Reverse Transcriptase PCR

**DOI:** 10.3390/ani10101806

**Published:** 2020-10-05

**Authors:** Keren E. Dittmer, Rosemary W. Heathcott, Jonathan C. Marshall, Sara Azarpeykan

**Affiliations:** 1 School of Veterinary Science, Massey University, Palmerston North 4410, New Zealand; r.heathcott@xtra.co.nz (R.W.H.); sara.azarpeykan@newcastle.edu.au (S.A.); 2 School of Fundamental Sciences, Massey University, Palmerston North 4410, New Zealand; j.c.marshall@massey.ac.nz

**Keywords:** phosphatonin, fibroblast growth factor 23, phosphorus, kidney, sodium-phosphate co-transporter, NPT, parathyroid hormone, vitamin D

## Abstract

**Simple Summary:**

Traditionally, it has been thought that control of body phosphorus was secondary to the tighter control of calcium. However, over the last 20 years, an extensive system for control of body phosphorus by proteins called phosphatonins has been shown to exist. Most research on phosphatonins has been done in rat or mouse models. This paper looks at whether important proteins and phosphorus channels in the phosphatonin pathways are present in the kidneys of dogs, horses and sheep. The results showed that all of the components of the phosphatonin system are present in these species, but that there are species differences in which protein or channel is most common, and in the relationships between the proteins and channels. This research is important because the phosphatonin system is involved in the progression of chronic kidney disease in humans and animals, and differences in the systems between animal species may affect treatment of chronic kidney disease.

**Abstract:**

The aim of this preliminary study was to determine the relative expression of phosphatonin pathway-related genes in normal dog, sheep and horse kidneys and to explore the relationships between the different genes. Kidneys were collected post-mortem from 10 sheep, 10 horses and 8 dogs. RNA was extracted, followed by reverse transcriptase quantitative polymerase chain reaction for fibroblast growth factor receptor 1 IIIc (*FGFR1IIIC*), sodium-phosphate co-transporter (*NPT*) 1 (*SLC17A1*), *NPT2a* (*SLC34A1*), *NPT2c* (*SLC34A3*), parathyroid hormone 1 receptor (*PTH1R*), klotho (*KL*), vitamin D receptor (*VDR*), 1a-hydroxylase (*CYP27B1*) and 24-hydroxylase (*CYP24A1*). *NPT2a* was highly expressed in the dog kidneys, compared with those of the horses and sheep. *NPT1* had greatest expression in horses and sheep, although the three different NPTs all had relatively similar expression in sheep. There was little variability in *FGFR1IIIc* expression, particularly in the dogs and horses. *FGFR1IIIc* expression was negatively correlated with *NPT* genes (except *NPT2a* in sheep), while *NPT* genes were all positively correlated with each other. Unexpectedly, klotho was positively correlated with *NPT* genes in all three species. These results provide the basis for further research into this important regulatory system. In particular, species differences in phosphatonin gene expression should be considered when considering the pathogenesis of chronic kidney disease.

## 1. Introduction

The traditional paradigm of body phosphorus control has been that regulation of phosphorus was secondary to the tighter control of calcium. However, research over the last 20 years has shown that there is an extensive regulatory system to control body phosphorus by proteins known as phosphatonins [1]. This system is centered on fibroblast growth factor 23 (FGF23), a hormone that is produced by osteocytes in response to high plasma phosphorus concentrations [2]. In the kidneys, FGF23 forms a complex with α-klotho (klotho) and fibroblast growth factor receptor 1c (FGFR1c); limited binding to FGFR3 and -4 may also occur [3,4]. This leads to downregulation of sodium-phosphate co-transporters (NPT2a and 2c) and, subsequently, increased renal phosphorus excretion [5].

The other major effect of FGF23 on plasma phosphorus concentration is via inhibition of 25-hydroxyvitamin D 1α-hydroxylase and, possibly, activation of 25-hydroxyvitamin D 24-hydroxylase [6,7]. Additionally, binding of FGF23 to the klotho-FGFR1c complex in the parathyroid gland results in decreased parathyroid hormone (PTH) secretion [8].

FGF23 is a key factor in the pathogenesis of chronic kidney disease–metabolic bone disease, where significant increases in plasma FGF23 concentrations occur in humans, cats and dogs in parallel with the stages of kidney disease [9,10,11]. Plasma FGF23 concentrations in humans and animals with chronic kidney disease are also correlated with serum phosphorus concentrations, and, in some studies, with plasma PTH concentrations [9,12,13].

Species differences in phosphorus metabolism have been described. In mice, NPT2a (SLC34A1) is considered to be the major sodium phosphate co-transporter, with NPT2c (SLC34A3) playing a minor role [14]. The opposite appears to be the case in humans [15,16]. However, while the expression of phosphatonin-related proteins is relatively well described in the kidneys of goats [17,18,19] and in the intestines of horses, goats and cattle [20,21,22,23], knowledge on the expression of phosphatonin-related proteins in the kidneys of most domestic species is lacking.

The aim of this preliminary study was to determine the relative expression of phosphatonin pathway-related genes in normal dog, sheep and horse kidneys and to explore the relationships between the different genes. In addition, we aimed to compare the patterns of expression and gene relationships between species. These species were chosen to cover a wide range of evolutionary adaptations, for example, carnivores, monogastric herbivores and ruminants.

## 2. Materials and Methods

Kidney samples were collected post-mortem from 10 healthy adult horses, 1–14 years of age (6 male neutered, 2 male, 2 female; 5 Thoroughbreds, 5 Standardbreds), 10 healthy adult sheep, 4–6 years of age (all female, all Romney), and eight healthy dogs, 1–2 years of age (5 female, 3 male; 1 Neapolitan Mastiff, 2 Staffordshire bull terrier cross, 5 Pit bull terrier cross) that were euthanized for reasons unrelated to this study. The horses had been kept at pasture and supplemented with hay prior to euthanasia; the sheep were also kept at pasture prior to euthanasia; the dogs were fed the same commercial dog kibble for at least 2 weeks prior to euthanasia. As the kidney collection was post-mortem and unrelated to the reasons for euthanasia, animal ethics approval was not required as per national guidelines.

Kidney samples were collected within half an hour of euthanasia, divided into two, with one sample either snap-frozen in liquid nitrogen or placed into Ambion RNAlater (Thermo Fisher Scientific Inc., Waltham, MA, USA), and the other placed into 10% neutral buffered formalin and processed for histology. Hematoxylin and eosin-stained sections were examined by the primary author to confirm the absence of significant lesions. Samples for qPCR were stored at −80 °C until processing.

RNA was extracted using Tri Reagent (Sigma-Aldrich Inc., Merck KGaA, Darmstadt, Germany) as per the manufacturer’s instructions. RNA was treated with the Ambion Turbo DNA-free kit (Thermo Fisher Scientific Inc., Waltham, MA, USA) to remove any contaminating genomic RNA as per the manufacturer’s instructions. RNA and DNA concentrations (to check for absence of DNA) were measured using the Qubit 2.0 Fluorometer with Qubit RNA HS (high sensitivity), and DNA HS assays (Thermo Fisher Scientific Inc., Waltham, MA, USA). In addition, RNA integrity was assessed by running 5 μL of the RNA sample on a 1.5% agarose gel containing SYBR Safe (Thermo Fisher Scientific Inc., Waltham, MA, USA) with visualization of sharp 28S and 18S bands using an ultraviolet transilluminator.

The Transcriptor First Strand cDNA Synthesis Kit (Roche, Mannheim, Germany) was used to synthesize cDNA. Each 20 μL reaction mix contained 600 ng of RNA, 2.5 μM oligo (dT), 8 mM RT (reverse transcriptase) reaction buffer, 1 mM dNTP, 10 U reverse transcriptase, 20 U RNase inhibitor and RNase/DNase-free water. The reaction was performed at 55 °C for 30 min, 85 °C for 5 min, and then chilled at 4 °C using an Applied Biosystems Veriti Thermal Cycler (Thermo Fisher Scientific Inc., Waltham, MA, USA).

Primers were designed using Geneious 8.1 (Biomatters Ltd., Auckland, New Zealand) and the Primer 3 algorithm, as described previously [24]. The primers were designed with the following features: PCR product less than 150 bp, span an exon–exon junction, and show no complementarity to unrelated targets. Each primer set was tested for secondary structures at 60 °C using the mFold program (http://mfold.rit.albany.edu).

Real-time qPCR was performed using the StepOne Plus real-time PCR machine (Applied Biosystems, Thermo Fisher Scientific Inc., Waltham, MA, USA). Each real-time PCR reaction mix contained 5 μL Power SYBR Green PCR master mix (Thermo Fisher Scientific Inc., Waltham, MA, USA), the primer pair at concentrations determined to be optimal, as in Tables S1–S3, and 10 ng of cDNA, made up to 10 μL with RNase/DNase-free water. The PCR protocol consisted of a denaturation step at 95 °C for 20 s, followed by 40 cycles at 95 °C for 3 s and 60 °C for 30 s, and a melt curve ranging from 60 °C to 95 °C with a heating rate of 0.3 °C/15 s. Negative controls of water and reaction mix without reverse transcriptase were included in every PCR run, and all samples were run in duplicate. Standard curves were produced for each gene target in order to determine the efficiency and R^2^ (regression coefficient).

All samples were normalized relative to the expression of the same housekeeping gene found to be most stable in the kidneys of all three species: zeta polypeptide (*YWHAZ*) [24,25,26].

Statistical analysis was performed as previously described [24] using R-studio 1.1.456 with R version 3.5.1 (R Core Team, 2016) [27] and the lme4 package [28].

## 3. Results

**Figure 1:**
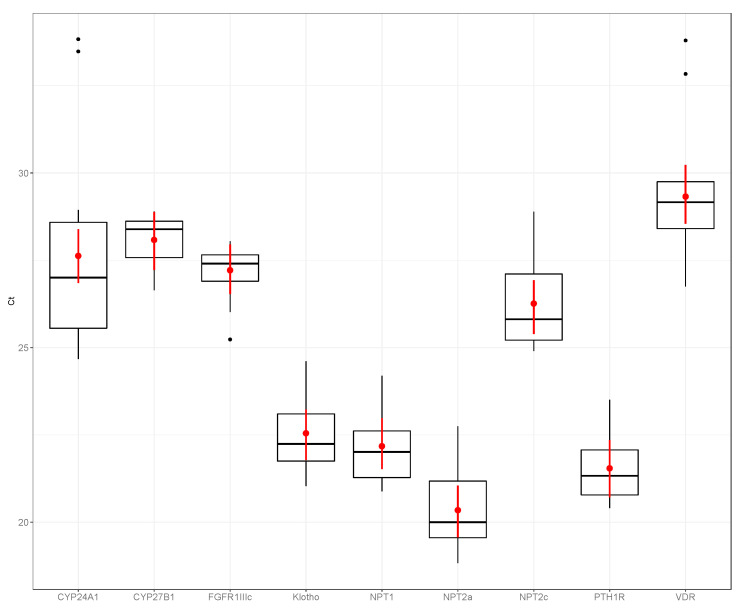
Expression of phosphatonin and selected vitamin D-related transcripts in canine kidney—cytochrome P450 family 24 subfamily A polypeptide 1 (*CYP24A1*), cytochrome P450 family 27 subfamily B polypeptide 1 (*CYP27B1*), fibroblast growth factor receptor 1 IIIc (*FGFR1IIIc*), α-klotho (klotho, *KL*), sodium-phosphate co-transporter 1 (*NPT1*), *NPT2a*, *NPT2c*, parathyroid hormone 1 receptor (*PTH1R*) and vitamin D receptor (*VDR*). Boxplots are normalized cycle threshold numbers (Ct values), where the median expression levels of genes are shown as black bars, the red dots show the mean expression of genes and the red line bars represent 95% confidence interval of the genes, accounting for replicates.

**Figure 2:**
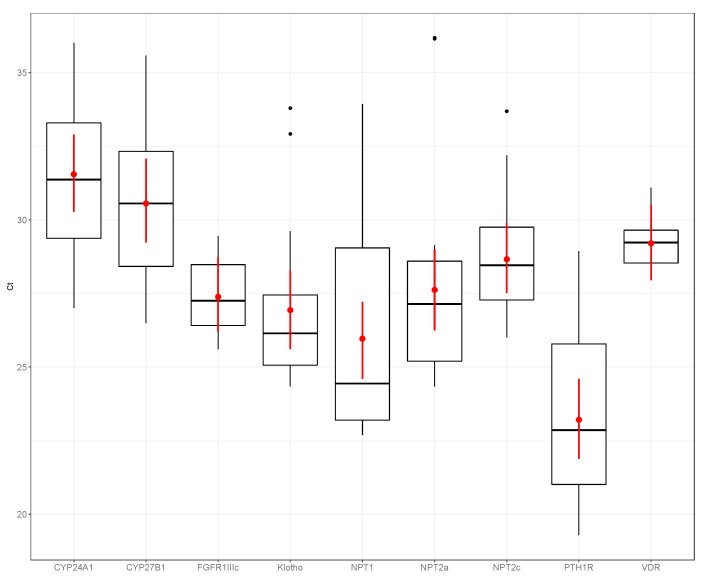
Expression of phosphatonin and selected vitamin D-related transcripts in ovine kidneys—cytochrome P450 family 24 subfamily A polypeptide 1 (*CYP24A1*), cytochrome P450 family 27 subfamily B polypeptide 1 (*CYP27B1*), fibroblast growth factor receptor 1 IIIc (*FGFR1IIIc*), α-klotho (klotho, *KL*), sodium-phosphate co-transporter 1 (*NPT1*), *NPT2a*, *NPT2c*, parathyroid hormone 1 receptor (*PTH1R*) and vitamin D receptor (*VDR*). Boxplots are normalized cycle threshold numbers (Ct values), where the median expression levels of genes are shown as black bars, the red dots show the mean expression of genes and the red line bars represent 95% confidence interval of the genes, accounting for replicates.

**Figure 3:**
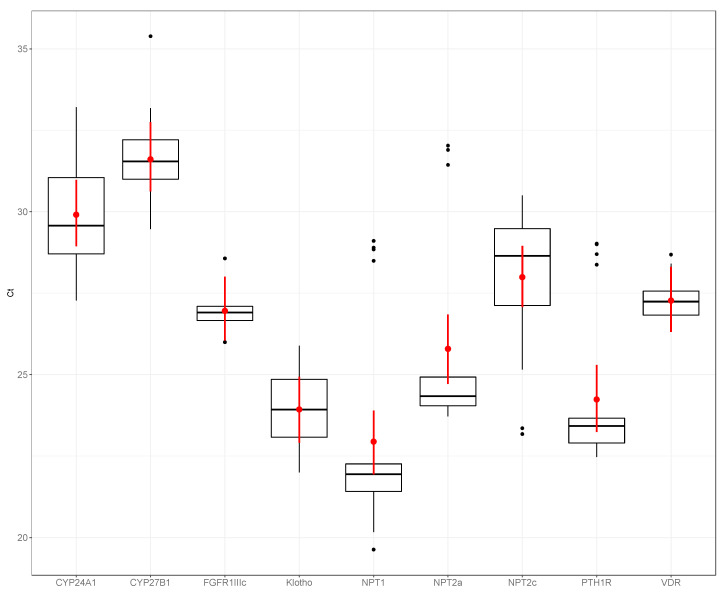
Expression of phosphatonin and selected vitamin D-related transcripts in equine kidneys—cytochrome P450 family 24 subfamily A polypeptide 1 (*CYP24A1*), cytochrome P450 family 27 subfamily B polypeptide 1 (*CYP27B1*), fibroblast growth factor receptor 1 IIIc (*FGFR1IIIc*), α-klotho (klotho, *KL*), sodium-phosphate co-transporter 1 (*NPT1*), *NPT2a*, *NPT2c*, parathyroid hormone 1 receptor (*PTH1R*) and vitamin D receptor (*VDR*). Boxplots are normalized cycle threshold numbers (Ct values), where the median expression levels of genes are shown as black bars, the red dots show the mean expression of genes and the red line bars represent 95% confidence interval of the genes, accounting for replicates.

Figure 1, Figure 2 and Figure 3 show the expression of the different phosphatonin pathway genes examined in the dogs, sheep and horses. Expression of *FGFR1c* showed little variability, particularly in the dogs (range count threshold (Ct) 25.23–28.04) and horses (range Ct 25.99–28.57). *NPT2a* expression in the dogs was high (range Ct 18.82–22.74) compared with the sheep (range Ct 24.34–36.18) and horse (range Ct 23.72–32.02). Of the sodium-phosphate cotransporters, *NPT1* had the greatest expression in the horses and sheep, and *NPT2a* in the dogs. Although expression of all the sodium-phosphate cotransporters in the sheep was similar. *NPT2c* expression was the lowest of all the cotransporters in all three species.

**Figure 4:**
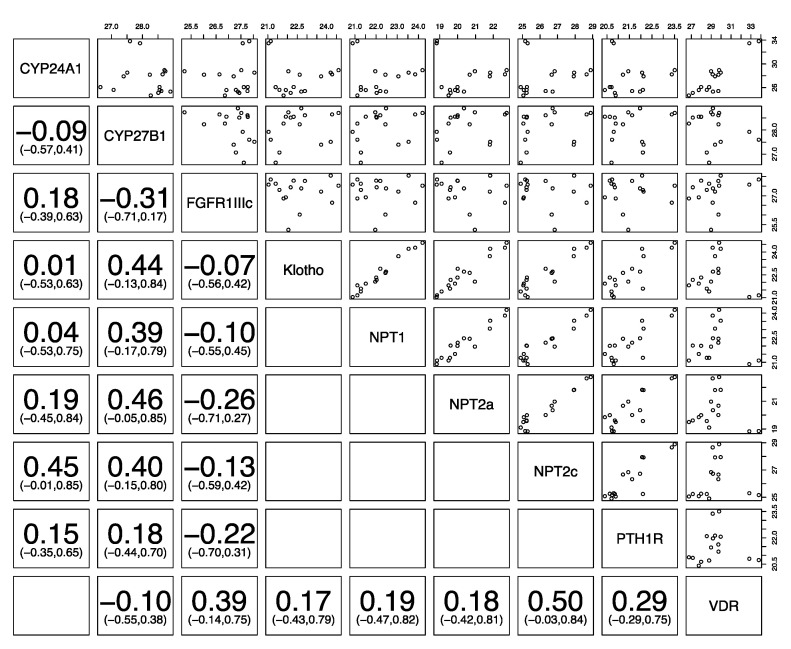
Correlation of phosphatonin and selected vitamin D-related transcripts in canine kidneys. Spearman’s correlation (95% confidence intervals) between cytochrome P450 family 24 subfamily A polypeptide 1 (*CYP24A1*), cytochrome P450 family 27 subfamily B polypeptide 1 (*CYP27B1*), fibroblast growth factor receptor 1 IIIc (*FGFR1IIIc*), α-klotho (klotho, *KL*), sodium-phosphate co-transporter 1 (*NPT1*), *NPT2a*, *NPT2c*, parathyroid hormone 1 receptor (*PTH1R*) and vitamin D receptor (*VDR*). Statistically significant positive and/or negative correlations of different genes with each other (*p* < 0.05) are bolded, and the numbers in parentheses show the 95% confidence interval. On the top and right, the numbers indicate normalized gene expression.

**Figure 5:**
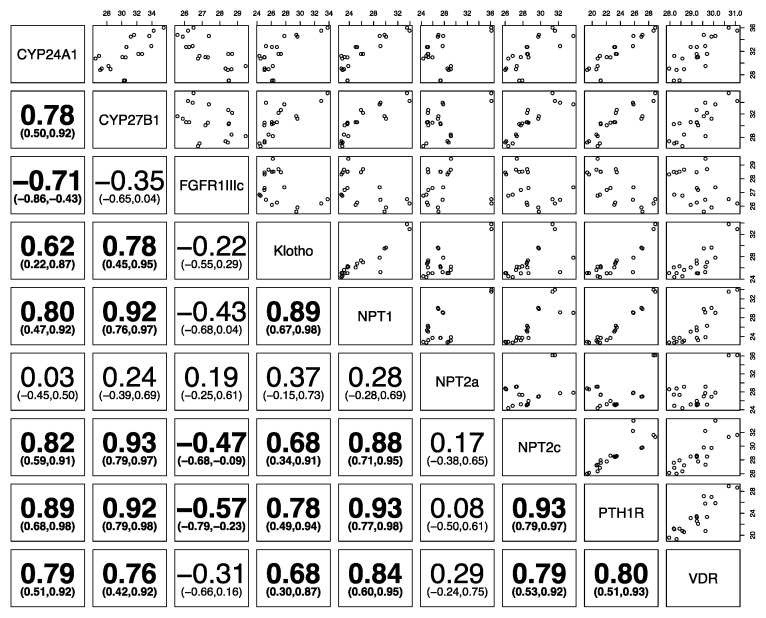
Correlation of phosphatonin and selected vitamin D-related transcripts in ovine kidneys. Spearman’s correlation (95% confidence intervals) between cytochrome P450 family 24 subfamily A polypeptide 1 (*CYP24A1*), cytochrome P450 family 27 subfamily B polypeptide 1 (*CYP27B1*), fibroblast growth factor receptor 1 IIIc (*FGFR1IIIc*), α-klotho (klotho, *KL*), sodium-phosphate co-transporter 1 (*NPT1*), *NPT2a*, *NPT2c*, parathyroid hormone 1 receptor (*PTH1R*) and vitamin D receptor (*VDR*). Statistically significant positive and/or negative correlations of different genes with each other (*p* < 0.05) are bolded, and the numbers in parentheses show the 95% confidence interval. On the top and right, the numbers indicate normalized gene expression.

**Figure 6:**
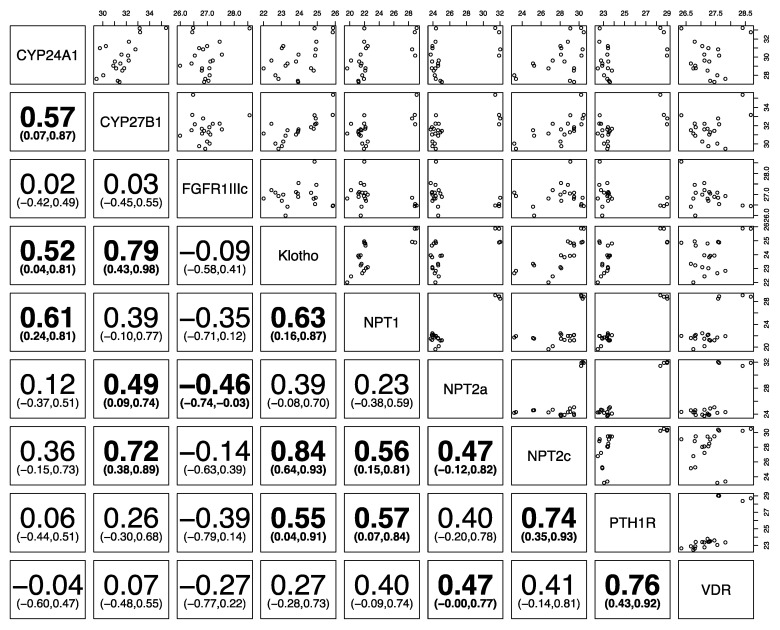
Correlation of phosphatonin and selected vitamin D-related transcripts in equine kidneys. Spearman’s correlation (95% confidence intervals) between cytochrome P450 family 24 subfamily A polypeptide 1 (*CYP24A1*), cytochrome P450 family 27 subfamily B polypeptide 1 (*CYP27B1*), fibroblast growth factor receptor 1 IIIc (*FGFR1IIIc*), α-klotho (klotho, *KL*), sodium-phosphate co-transporter 1 (*NPT1*), *NPT2a*, *NPT2c*, parathyroid hormone 1 receptor (*PTH1R*) and vitamin D receptor (*VDR*). Statistically significant positive and/or negative correlations of different genes with each other (*p* < 0.05) are bolded, and the numbers in parentheses show the 95% confidence interval. On the top and right, the numbers indicate normalized gene expression.

Figure 4, Figure 5 and Figure 6 show the correlation of the expression of the different phosphatonin pathway genes with each other. Klotho was positively correlated in all species with the sodium-phosphate co-transporters *NPT1*, *NPT2a* and *NPT2c* and *PTH1R*. *FGFR1IIIc* was negatively correlated with klotho, the sodium-phosphate co-transporters *NPT1*, *NPT2a*, and *NPT2c* and *PTH1R* in all species, except *NPT2a* in sheep. Similarly, *PTH1R* was positively correlated with all genes in the three species (except *FGFR1IIIc* in all species). The sodium-phosphate co-transporters *NPT1*, *NPT2* and *NPT2c* were positively correlated with each other in all species.

## 4. Discussion

The results show that phosphatonin system genes are expressed in the kidney of dogs, sheep and horses. Each species showed a different pattern of sodium-phosphate co-transporter gene expression. The sodium-phosphate co-transporters were all positively correlated with each other in the 3 species. *NPT2a* had profoundly greater expression in dogs than in sheep and horses, as shown by the mean Ct value of 20.4 in the dog, compared with 27.6 and 25.8 in the sheep and horse respectively. The dog, as a carnivore, has a higher dietary phosphorus consumption in comparison to herbivores such as the horse and sheep [29]. Therefore, higher expression of *NPT2a* mRNA could be an evolutionary adaptation to allow for greater renal phosphorus excretion.

NPT2a is considered to be the predominant sodium-phosphate co-transporter in the kidneys of mice, although NPT2c is considered the predominant human co-transporter. *NPT2c* expression was the lowest of all the co-transporters in the three species examined. In mice, *NPT2* was found to account for 84% of total Na-P co-transporter expression, with *NPT1* expression accounting for 15% [30]. In horses and sheep, *NPT1* had the greatest expression of the sodium-phosphate co-transporters (although all three co-transporters had relatively similar expression in the sheep).

NPT1, when first discovered, was thought to be primarily a phosphate transporter; however, further study has revealed that, in addition to phosphate, it transports a variety of different anions, in particular urates [31]. PTH was found to have no effect on renal phosphate transport in *Npt2-/-* mice [32], suggesting there is a role for NPT1 in renal phosphate excretion. However, expression of both *NPT1* and *NPT2* was significantly decreased in the *Hyp* mouse (a mouse model of hypophosphatemic rickets with increased serum FGF23 concentrations) [30], and *NPT1* expression is upregulated in *Npt2-/-* mouse models [33]. In this present study, expression of *NPT1* was correlated with that of *NPT2a*, *NPT2c* and *PTH1R* in the three species examined. This could suggest species differences in the function and control of NPT1. High *NPT1* expression in horses could be a physiological adaptation. Plasma uric acid concentrations in horses increase with intense exercise [34] and endurance exercise [35], and perhaps high renal *NPT1* expression may assist the horse with removal of uric acid produced during exercise. Similarly, a study examining the kinetic parameters of sodium-phosphate co-transporter IIa in sheep and goats found that dietary phosphorus or calcium deficiency did not alter NPT2a transport capacity or affinity [36]. The authors suggested that PTH-independent pathways for phosphorus reabsorption may be important in the kidneys of ruminants, perhaps suggesting a role for NPT1 in renal tubular phosphate transport in sheep and goats.

Binding of membrane-bound klotho to the FGFR1c receptor activates the ERK1/2 pathway, resulting in decreased numbers of sodium-phosphate co-transporters in the membrane of proximal tubular epithelial cells [3]. Therefore, it could be expected that klotho and *FGFR1c* would be negatively correlated with renal sodium-phosphate co-transporters. This expectation holds true for *FGFR1c* in all species, where there is a negative association with the sodium-phosphate co-transporters, with the exception of *NPT2a* in the sheep.

However, klotho is significantly positively associated with sodium-phosphate co-transporters in all three species examined, a result that is inconsistent with knowledge on the regulation of sodium-phosphate co-transporters. Klotho does, however, have many other functions, including an association with aging [37]. Klotho is also involved in renal calcium and potassium transport and control of insulin-like growth factor-1 receptors and transforming growth factor-β1 [38,39,40,41]. Therefore, klotho gene expression in this study could be related to its other functions rather than its action on sodium-phosphate co-transporters. Expression of klotho in the body is highest in the kidneys and two forms may be produced—membrane bound klotho and secreted klotho—depending on RNA splicing. The design of the PCR primers would have detected both forms of klotho, perhaps also explaining the discrepancy in the relationship between klotho and the sodium-phosphate co-transporters.

Expression of *FGFR1c* showed little variability, particularly in the dogs and horses, despite the age and breed diversity of these two groups. FGFR1c is one of a group of four FGF receptors (FGFR1–4), and FGFR1 has three isoforms (a–c) with different binding affinities for different FGFs [42]. FGF23 can bind to FGFR1, 3 and 4, but signaling is maximum when bound to FGFR1c complexed to klotho [4]. There is limited literature on the regulation of FGFR1 receptors in the kidneys and on the relationship between *FGFR1* and other phosphatonin pathway genes.

*FGFR1c* was significantly negatively correlated with *CYP27B1* gene expression in sheep and dogs (but not horses), again likely reflecting the action of FGF23 in suppression of CYP27B1 [43]. Conversely, the relationship between *FGFR1c* and *CYP24A1* was the opposite in sheep as to what might have been expected. There is some suggestion that FGF23 may increase *CYP24A1* expression [44] as part of its role in decreasing plasma 1,25(OH)_2_D concentrations. However, whether it has a direct role in this is controversial, as 1,25(OH)_2_D itself is a potent inhibitor of *CYP24A1* and separating the actions of FGF23 on 1,25(OH)_2_D and *CYP24A1* is difficult. Other studies have found no effect of FGF23 on *CYP24A1* gene expression [45]. Renal expression of *VDR* and *CYP24A1* was significantly correlated in the sheep and dogs in this study, and this has also been reported in other studies [24]. These results suggest that control of *CYP24A1* expression in the sheep may be indirect, due to decreased production of 1,25(OH)_2_D rather than a direct action of FGF23 itself.

*PTH1R* was highly expressed in all three species and had a positive correlation with all three sodium-phosphate co-transporters in all three species. Activation of PTH1R by PTH leads to inhibition of NPT2a, counter to the findings in our study. However, as with klotho, the regulation of PTH1R and the sodium-phosphate co-transporters is more complex than a simple two-way relationship. For example, PTH1R may couple with a number of different stimulatory G proteins, which may activate different secondary messenger pathways, and PTH1R on the apical and basolateral membranes of the renal tubular epithelial cell may have different roles [46]. In addition, the function of PTH1R on sodium-phosphate co-transporter expression in renal tubular epithelial cells is modulated by the binding of PTH1R to NHERF1 (sodium hydrogen exchanger regulatory factor 1) and activation of the phospholipase C pathway, which ultimately leads to NPT2a internalization from the cell membrane [47,48]. As such, there are a number of physiological intermediates between PTH1R and the sodium-phosphate co-transporters, suggesting that direct correlation may not be appropriate.

The major limitation of this study is the lack of concurrent data on plasma phosphorus and calcium concentrations. In addition, the control of calcium-phosphorus homeostasis by FGF23, 1,25OH_2_D_3_ and PTH is so inter-linked that determining relationships in isolation should be treated with caution. Therefore, future studies into the phosphatonin system should also assess other calciotropic genes, such as calcium channels and binding proteins, and assess intermediary cell messengers, such as NHERF, phosphokinase A and phospholipase C pathways. An additional limitation of the study is that the kidney samples were collected from animals of different age, sex and breed, and it is possible these differences may have resulted in the observed changes in gene expression. In particular, the wide age range in the horse samples could have contributed to the greater variation seen in gene expression in this species.

In summary, phosphatonin pathway genes are expressed in the kidneys of dogs, sheep and horses. *NPT2a* expression was greater in dogs compared with sheep and horses, and *NPT2c* expression was the lowest of all the sodium-phosphate transporters in all species. There was little variation in *FGFR1c* expression, suggesting that it may be tightly regulated, and *FGFR1c* was negatively correlated with the sodium-phosphate co-transporters. Due to the variability in expression and relationships between the different phosphatonin genes in the different species in this study, species differences in phosphatonin pathway regulation should be considered when investigating the pathogenesis of and possible treatments for chronic kidney disease. Additionally, further studies investigating the effect of sex and age would also be warranted.

## Supplementary Materials

The following are available online at https://www.mdpi.com/2076-2615/10/10/1806/s1, Table S1: Canine phosphatonin and selected vitamin D-related kidney genes primer sequences and concentrations, Table S2: Ovine phosphatonin and selected vitamin D-related kidney genes primer sequences and concentrations, Table S3: Equine phosphatonin and selected vitamin D-related kidney genes primer sequences and concentrations.
