# Supplementary material for: Expression of Phosphatonin-Related Genes in Sheep, Dog and Horse Kidneys Using Quantitative Reverse Transcriptase PCR
Source: Animals (Basel). 2020 Oct 5;10(10):1806. doi: 10.3390/ani10101806 (PMC7601102; doi:10.3390/ani10101806)
Supplement: Supplementary file 1 [file animals-10-01806-s001.pdf]

Supplementary Table 1. Canine phosphatonin and selected vitamin D-related kidney genes - cytochrome P450 family 24 subfamily A polypeptide 1 (CYP24A1), cytochrome P450 family 27 subfamily B polypeptide 1 (CYP27B1), fibroblast growth factor receptor 1 IIIc (FGFR1IIIc), α-klotho (klotho), sodium-phosphate co-transporter 1 (NPT1), NPT2a, NPT2c, parathyroid hormone 1 receptor (PTH1R), vitamin D receptor (VDR), and reference gene zeta polypeptide (YWHAZ).

**Gene**	**Primer (5′-3′)**	**Amplicon length (bp)**	**Primer concentration (nM)**	**PCR efficiency (%)**	**Regression coefficient (R^2)**
CYP24A1 [1]	F: GAGGCCGCATTGAAGACTTA<br>R: CATTCTTCCGAAGGAGTCCA	102	450:450	94.7	0.98
CYP27B1 [1]	F: GAGCTGCAAATGGCTTTGGCTCAG<br>R: CTGTAGGTTGATGCTCCTCTCGGG	123	300:300	89.2	0.99
FGFR1IIIc	F: GTATACGTGCTTGGCGGGTA<br>R: AACGGTCAACCATGCAGAGT	61	300:300	96.9	0.99
Klotho	F: CCTGTGACTTTGCTTGGGGA<br>R: CAGCCCGTCCACCTTAATCA	131	900:900	95.5	0.99
NPT1 (SLC17A1)	F: GGCAATCAAGAGCACACACG<br>R: TTCTTCCTTGGGACGCTGAC	135	900:900	98.2	0.99
NPT2a (SLC34A1)	F: CAGGTGGCTAAGGTCATTCAGA<br>R:GGGGTGATGGCTGAAGTGAA	146	300:300	96.5	0.99
NPT2c (SLC34A3)	F: CATCTGCTCCCTGGACATCC<br>R: TCTGCACGAGAACTGTGACC	143	300:300	90.9	0.98
PTH1R	F: TCATCAACATCGTCCGGGTG<br>R: TTGAGCAGCTTCCGGTACTG	91	900:900	95.0	0.99
VDR [1]	F: AGCATCCAAAAGGTCATTGG	92	900:800	95.2	0.97

	R: GCACTTGATTTCAGCAGCAC				
YWHAZ	F: AGCAGAGAGCAAAGTCTTCTATT<br>R: GACTGATCCACAATCCCTTTCT	101	900:900	91.4	1.00

[1](Azarpeykan et al., 2016 b)

Supplementary Table 2. Ovine phosphatonin and selected vitamin D-related kidney genes - cytochrome P450 family 24 subfamily A polypeptide 1 (CYP24A1), cytochrome P450 family 27 subfamily B polypeptide 1 (CYP27B1), fibroblast growth factor receptor 1 IIIc (FGFR1IIIc), α-klotho (klotho), sodium-phosphate co-transporter 1 (NPT1), NPT2a, NPT2c, parathyroid hormone 1 receptor (PTH1R), vitamin D receptor (VDR), and reference gene zeta polypeptide (YWHAZ).

**Gene**	**Primer (5′-3′)**	**Amplicon length (bp)**	**Primer concentration (nM)**	**PCR efficiency (%)**	**Regression coefficient (R^2)**
CYP24A1 [1]	F: CTGTGATGAGAGAGGCCGCATTGA<br>R: AGCTTCCTCCCCTGCCTTCTT	128	600:600	103.9	0.99
CYP27B1 [1]	F: GCAGAGCTTGAGTTGCACAT<br>R: CTTCTCTCAGGCACCAGGAC	119	250:250	102.2	0.92
FGFR1IIIc	F: ACACCACCGACAAAGAGATGG<br>R: TCAGCCATGCAGAGTGATGG	120	900:900	99.3	0.99
Klotho	F: TGTGGAGAATGGCTGGTTTGT<br>R: TACCCAATGACATCCACCCC	129	300:300	96.0	1.00
NPT1 (SLC17A1)	F: TCCCACTGGCAGCTGAATTT<br>R: CTCAGAGCAGTAAGTCGGCC	148	300:300	98.1	1.00
NPT2a (SLC34A1)	F: CACCATGACCCACTGCCTG<br>R: TCCAGGGAGCAGACGAAGAG	149	300:300	98.8	0.99
NPT2c (SLC34A3)	F: CGACATCCTCAAGGTGCTGA<br>R: AATAAGGCTCCGGTTGGTGG	109	300:300	100	0.99
PTH1R	F: CAGTACCGGAAGCTGCTCAA<br>R: TTGCCAGAGAGTCCCTGAGA	111	900:900	98.6	1.00
VDR [1]	F: TCATGCTGCGCTCCAACCAGT<br>R: TGGAACTTGATGAGGGGCTCGAT	140	400:400	93.5	0.98

YWHAZ	F: AGACGGAAGGTGCTGAGAAA<br>R: CGTTTGGGATCAAGAACTTT	123	300:300	101.7	0.98

[1](Azarpeykan et al., 2016 b)

Supplementary Table 3. Equine phosphatonin and selected vitamin D-related kidney genes - cytochrome P450 family 24 subfamily A polypeptide 1 (CYP24A1), cytochrome P450 family 27 subfamily B polypeptide 1 (CYP27B1), fibroblast growth factor receptor 1 IIIc (FGFR1IIIc), α-klotho (klotho), sodium-phosphate co-transporter 1 (NPT1), NPT2a, NPT2c, parathyroid hormone 1 receptor (PTH1R), vitamin D receptor (VDR), and reference gene zeta polypeptide (YWHAZ).

**Gene**	**Primer (5′-3′)**	**Amplicon length (bp)**	**Primer concentration (nM)**	**PCR efficiency (%)**	**Regression coefficient (R^2)**
CYP24A1 [1]	F:GTGTGATGAAAGAGGCCACATTGA<br>R: CGTTCTGCTGGAGGAGCCCG	113	350:350 nM	91.6	0.99
CYP27B1 [1]	F: CAGAGACATTCATGTGGGTGA<br>R: GCTGGACGAAAAGAATTTGG	117	300:300 nM	93.9	0.98
FGFR1IIIc	F: ACTGCCGGAGTTAATACCAC<br>R: CTTCCAGAACGGTCAACCA	145	300:300	101.7	0.99
Klotho	F: CCCAAGACAGGCTGAGAGTG<br>R: GCCGTGCGATCGTTAAATGA	121	300:300	102.3	0.98
NPT1 (SLC17A1)	F: TTGGTCTCCTAGCAGGTCAG<br>R: AGGCATACACAGAAGAGGGC	124	900:900	104.8	0.99
NPT2a (SLC34A1)	F: ATCATCACGGAGCCCTTCAC<br>R: GGAATAGTGGTGGGAGCCTC	149	300:300	99.0	0.99
NPT2c (SLC34A3)	F: CATCTGCTCCCTGGACATCC<br>R: TCTGCACGAGAACTGTGACC	143	900:900	106.4	0.99
PTH1R	F: CCTGCCATCTTCGTGACTGT<br>R: CACTTCTTGTTCCCGGAGCT	89	300:300	96.1	0.98
VDR[1]	F: ACAGCATCCAAAAGGTGGTC	89	500:500	99.9	0.99

	R: TGACTTCAGCAGCACGATCT				
YWHAZ [2]	F: TGTTGTAGGAGCCCGTAGGT<br>R: ATTCTCGAGCCATCTGCTGT	95	300:300	93.4	0.99

[1](Azarpeykan et al., 2016 b) [2](Azarpeykan et al., 2016 a)
